# Microarray Data Processing Techniques for Genome-Scale Network Inference from Large Public Repositories

**DOI:** 10.3390/microarrays5030023

**Published:** 2016-09-19

**Authors:** Sriram Chockalingam, Maneesha Aluru, Srinivas Aluru

**Affiliations:** 1 Department of Computer Science and Engineering, Indian Institute of Technology Bombay, Mumbai 40076, India; sriram.pc@iitb.ac.in; 2 School of Biology, Georgia Institute of Technology, Atlanta, GA 30332, USA; maneesha.aluru@biology.gatech.edu; 3 School of Computational Science and Engineering, Georgia Institute of Technology, Atlanta, GA 30332, USA

**Keywords:** microarray, gene networks, *Arabidopsis thaliana*

## Abstract

Pre-processing of microarray data is a well-studied problem. Furthermore, all popular platforms come with their own recommended best practices for differential analysis of genes. However, for genome-scale network inference using microarray data collected from large public repositories, these methods filter out a considerable number of genes. This is primarily due to the effects of aggregating a diverse array of experiments with different technical and biological scenarios. Here we introduce a pre-processing pipeline suitable for inferring genome-scale gene networks from large microarray datasets. We show that partitioning of the available microarray datasets according to biological relevance into tissue- and process-specific categories significantly extends the limits of downstream network construction. We demonstrate the effectiveness of our pre-processing pipeline by inferring genome-scale networks for the model plant *Arabidopsis thaliana* using two different construction methods and a collection of 11,760 Affymetrix ATH1 microarray chips. Our pre-processing pipeline and the datasets used in this paper are made available at http://alurulab.cc.gatech.edu/microarray-pp.

## 1. Introduction

Reverse engineering of gene networks from gene expression data is an active research area in systems biology (see [1] for a recent review). Such reverse-engineered networks provide biologists with a wealth of information on the relationships between genes and gene products. Clearly, the quality of generated networks is directly related to the size and quality of input data. With respect to the size of input data, the large number of publicly available experiments has been sufficient for network construction methods, and this has only increased over the past decade. However, when the input data is collected from public databases that contain the results of thousands of experiments conducted in hundreds of different labs around the world, a stringent pre-processing pipeline that includes appropriate quality control procedures is necessary.

Pre-processing methods designed for differential expression studies can also be used for the purpose of network inference. A recent review of the standard techniques used in pre-processing microarray data for differential expression studies can be found in Marbach et al. (2012) [2]. These pre-processing methods include quality control, normalization and filtering procedures. Quality control procedures are used to remove outlier microarray chips. Normalization procedures make it possible to compare experiments conducted under different conditions. Filtering procedures eliminate those genes whose expression values do not show significant variation. Many Bioconductor libraries have been developed to handle each of these three functions and are used as standard practices in microarray data analysis [3,4,5]. For a review of the best practices for selecting pre-processing methods for Affymetrix chips, see Florido et al. (2009) [6].

Most network construction methods adopt a pre-processing pipeline similar to the one described above. For example, standard methods applicable for Affymetrix ATH1 chips were used to re-construct *Arabidopsis thaliana* gene networks in Aluru et al. (2012) [7], Mao et al. (2009) [8] and Ma et al. (2007) [9]. However, when the number of input observations is in the order of tens of thousands, direct application of these procedures does not work as well. More specifically, we observe that these procedures tend to filter out a significant number of genes having a smaller dynamic range of expression, which in turn reduces the number of genes in the output networks. While such a reduction is convenient in differential expression studies, in the case of gene networks it reduces the discoverability of relationships of these missing genes. For example, in the construction of a correlation network for *Arabidopsis thaliana* reported in Mao et al. (2009) [8], the pre-processing pipeline selected only 16,293 genes for network construction, and the final network had only 6206 genes. Considering that *Arabidopsis thaliana* has more than 22,000 genes, this is a considerable loss in the number of genes selected for network construction, thus reducing the utility of reverse-engineered networks on a genome scale. Elimination of genes during pre-processing is not an indicator of the deficiency in the filtering process itself, but of the diverse nature of large datasets. Therefore, simply reducing the threshold values in filtering procedures cannot overcome this loss of genes during pre-processing.

To address this problem, we introduce an alternate pre-processing method to handle large datasets and genes, and expand the scope of network analyses to include a much larger number of genes than is feasible with standard pre-processing pipelines. While the method(s) introduced in this paper are applicable in general, we focus on Affymetrix ATH1 chips for *Arabidopsis* *thaliana*, a model plant with more than 22,000 genes and 35,000 proteins. Since it is a well-studied model plant, thousands of Affymetrix ATH1 microarray datasets are available for download from public databases. We demonstrate that the categorization of experiments into tissue- and process-specific categories, construction of genome-scale networks for each category, and generating their union network leads to the inclusion of a large number of genes and the uncovering of many more interactions than directly processing the entire dataset. Through this approach, we construct the largest microarray data generated *Arabidopsis* network to date that includes 20,126 genes, closer to the total number of genes for this organism. We also have made available the source code for our pre-processing pipeline at the URL: http://alurulab.cc.gatech.edu/microarray-pp.

This paper is organized as follows: In Section 2, we describe our methods for collecting datasets from public databases, removing duplicate data files, quality control, normalization, data filtering, and classification. In Section 3, we describe the results of the application of data filtering procedures on the classified datasets and compare them against conventional filtering techniques on the complete dataset. Finally in Section 4, we discuss the need for classification when using standard pre-processing procedures on large-scale microarray datasets.

## 2. Materials and Methods

### 2.1. Dataset Collection

We collected *Arabidopsis* Affymetrix ATH1 microarray datasets from three public repositories—ArrayExpress, Gene Express Omnibus (GEO) database and the now-defunct Nottingham Arabidopsis Stock Center (NASC) database. Each of these databases stores the experiment metadata in a different format, and therefore, we used different methods to collect data files from the corresponding websites. In case of GEO, we used the “GEOmetadb” Bioconductor library [10] to extract the description and experiment metadata, and used this metadata to download the corresponding microarray data files (CEL files) from National Center for Biotechnology Information (NCBI) web portal. For ArrayExpress, we downloaded the search results for Affymetrix ATH1 microarrays as a text file, which included the web URLs to the corresponding data files. We then parsed the text file and downloaded the CEL files corresponding to the search results. Downloading files from NASC database involved scrapping the HTML webpages related to the experiments and downloading the CEL files by following the appropriate HTML links.

We also added to our datasets collection Affymetrix ATH1 datasets prepared by Aluru et al. (2012) [7]. Since Aluru et al. (2012) [7] had collected all the data files submitted to public databases before 2010, we ignored all the database entries that were submitted prior to this date.

### 2.2. Removal of Duplicates and Bad CELs

Some experiments were found in multiple data repositories, potentially due to duplicative submissions to improve discoverability. We used the MD5 hash function to remove exact duplicate CEL files. We also noticed that some duplicate CEL files are not exact copies. However, the descriptions provided by submitter are highly similar in most cases. We inspected the CEL files for matching descriptions—first programmatically for exact matches, and then manually for uncovering highly similar descriptions and further inspection.

After eliminating all the duplicate CEL files, we manually scanned all the CEL files and removed those with irregular hybridization patterns.

### 2.3. Quality Control and Normalization

After the removal of duplicates, we filtered CEL files using standard quality control (QC) measures typical to the Affymetrix platform [6]. We used the “simpleaffy” BioConductor library [5] to inspect scale factors and presence of BioB spike-ins. Chips having more than three times the mean scale factor for a given submission or having no BioB spike-ins were removed.

We then removed outlier CEL files within each submission as follows. We computed “Relative Log Expression” (RLE) and “Normalized Unscaled Standard Errors” (NUSE) values within each submission. RLE and NUSE measures should be centered on zero and one, respectively. As a submission in a database is a set of CEL files, each CEL corresponding to a microarray assay conducted under different conditions by the same group, we expect an outlier CEL to have smaller spread of RLE and NUSE values. CEL files with interquartile range higher than 0.75 for RLE and NUSE were removed. Also, CEL files that were 0.075 from the required center for RLE and NUSE are identified as outliers and removed.

After the removal of CEL files that failed the above quality control procedures, we applied a normalization procedure so that gene expressions can be compared across experiments. We first converted the raw probe intensity values into expression values using the standard MAS 5.0 procedure with a scaling factor of 1000. Then, expression values are transformed to log2 space and mean centered, i.e., *G[i*, *j]* is updated to *G_i,j_*-*M_i_*, where *G_i,j_* is the raw expression value of gene *i* in chip *j*, *M_i_* is the average gene expression of gene *i* and *G[i*, *j]* is the normalized expression value of gene *i* in chip *j*. Finally, we used the “limma” Bioconductor library [4] to perform quantile normalization.

Table 1 shows the number of submissions, which we refer to as experiments in the rest of this paper, and the number of CEL files left after quality control procedures. Note that this table also includes 3546 CELs prepared by [7].

### 2.4. Data Filtering

In differential gene expression studies, where the main goal is to select genes with significant up or down regulation, genes with low expression range are eliminated because this indicates that those genes have no vital role in the pathway being studied. Filtering genes with low expression range is important in reverse-engineering gene networks also because most construction methods use correlation measures, and in order for a correlation measure between any two genes to be valid, the genes are expected to cover a wider expression range.

In the data filtering step, probes with lower expression range are identified using inter-quartile range (IQR) measure of the expression values. Typically, a threshold *q* is selected based on prior knowledge or empirical analysis, and all the probes having IQR < *q* is eliminated. In previous work where a gene network from a dataset of 3546 Affymetrix ATH CEL files was reported [7], an IQR threshold of 0.65 was selected. In Section 3.1, we discuss the effect of using this threshold on our dataset collection.

Clearly, lowering the value of the threshold q increases the number of genes that survive the filter, and consequently increase the size of the network. While it is desirable that the number of genes included in the network is as high as possible, simply reducing the IQR threshold to allow larger number of genes can produce spurious results in network construction due to invalid correlation measures, and thus undermining the primary goal of reverse-engineering networks.

A method for estimating the IQR threshold is expected to achieve two fundamental objectives:

(1) As demonstrated by Bourgona et al. (2010) [11], filtering of genes by a criterion independent of the statistical test used to detect the strongly differentiated genes increases the detection power of multiple testing. This is applicable for network construction methods also, because the valid correlations are evaluated using an interaction-by-interaction statistical testing.
(2) The set of genes that survive the filtering should reflect the dataset’s characteristic variability of expression values.

The method proposed by Kapetis et al. (2012) [12] to select the IQR threshold is based on [11] and is designed to accomplish the first objective. The method by Kapetis et al. (2012) [12] achieves this objective by identifying the probe-sets with the highest variability in signal across arrays. It computes the first derivative of the IQR profile and identifies its minimum point i.e., the steepest slope of the IQR profile plot.

**Figure 1:**
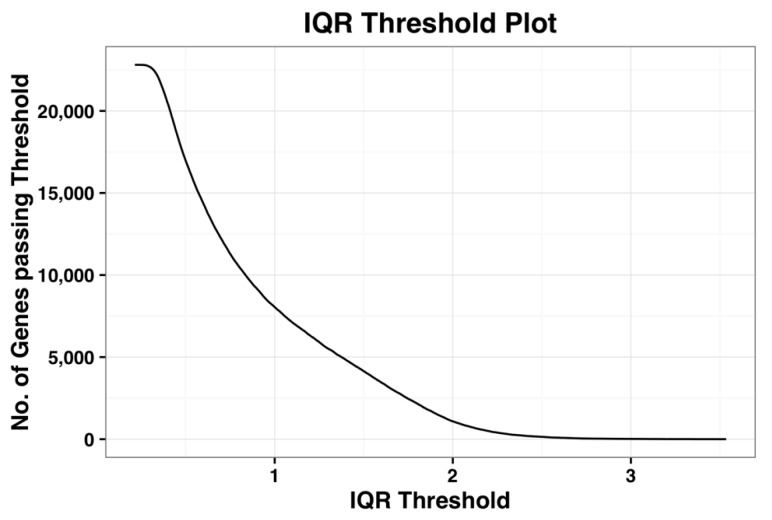
Plot of the inter-quartile range (IQR) profile of the complete dataset.

We now demonstrate this method by applying it on the complete dataset. Figure 1, shown below, is the plot of number of probe-sets that survive a given IQR threshold value. When the IQR threshold is zero, all the probe sets pass the threshold. As IQR increases, the number of genes that pass the filter reduces gradually. Kapetis et al. (2012) [12] call this plot as the IQR profile plot.

**Figure 2:**
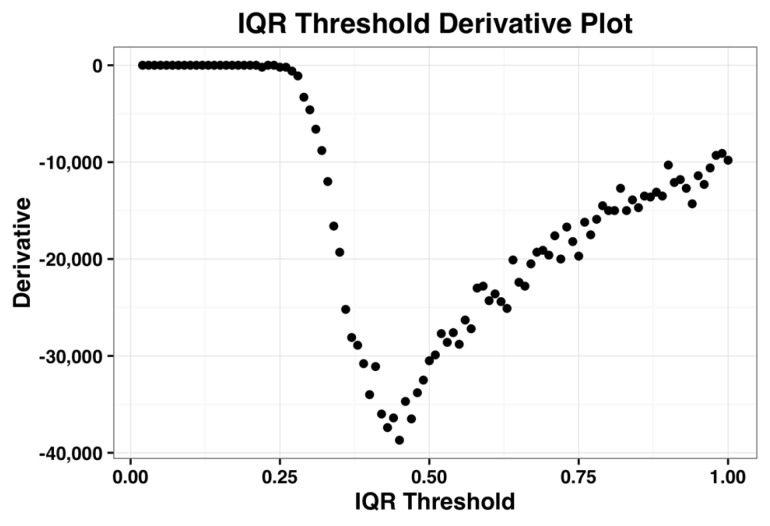
Plot of the first derivative of the inter-quartile range (IQR) profile for the complete dataset.

The derivative plot of the IQR profile of the complete dataset is shown in the Figure 2 below and its minimum occurs when IQR threshold is 0.44. Kapetis et al. (2012) [12] select this minimum value as the threshold. We refer the reader to [11,12] for further details on this method.

**Figure 3:**
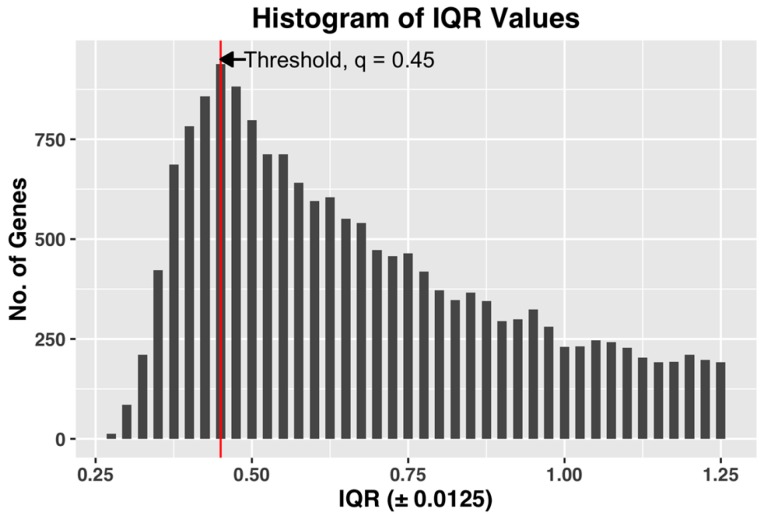
Histogram plot of IQR values of gene expression profiles for the entire dataset. For this dataset, an IQR of 0.45 ± 0.0125 shows the maximum value for number of genes, and hence the threshold *q* = 0.45.

We used the following method to estimate the threshold *q*, which achieves both of the desired objectives. After computing the gene expression IQR values of all the 22,810 probe-sets, we constructed a histogram plot of the 22,810 IQR values. Based on the plot, we selected *q* as the IQR value *x*, which has the maximum number of genes in the bin. The histogram bin size is selected to be granular enough to allow us to select a unique value of the threshold *q*. Figure 3, shown below, demonstrates the selection of threshold q by plotting the histogram plot of IQR values for a complete dataset of all the CEL files.

The selected q, by being the IQR value corresponding to the bin with maximum number of genes, i.e., the most common IQR expression value in the dataset, represents the characteristic IQR value of the dataset. Any value to the right of the selected *q* will reject the representative majority and can potentially leave out genes showing sufficient activity within the dataset.

We also found that in our datasets, the steepest slope of IQR plot always appears before the maximum point of the IQR histogram plot (See Figure 3 for example). Since the method by Kapetis et al. (2012) [12] uses the steepest slope of the IQR plot, the value q selected by our method is at least as high as the value selected by their method. Our method, while being simpler and easier to compute compared to Kapetis et al. (2012) [12], accomplishes both the objectives.

### 2.5. Probe Annotation

An Affymetrix ATH chip is a raw image containing the intensity values of 22,810 probe-sets. Due to continuing changes in annotation of the probe-sets of *Arabidopsis thaliana*, many probe-sets match with multiple genes and vice versa. We used the annotation files available with The Arabidopsis Information Resource (TAIR) [13] and Affymetrix [14], and created an initial map that contained all the 22,810 probe-sets. We first removed all the probes that were characterized as “no_match”, i.e., probes with no corresponding Arabidopsis Gene Identifier (AGI). Then, we used this map to remove those genes that map to more than three AGIs. Finally, we ran a clustering algorithm that placed two probes in the same cluster if they share at least one AGI. For each such cluster, a probe-set that mapped to the fewest number of AGIs was selected as its representative and all other probe-sets from the cluster were removed.

### 2.6. Classification

Our final dataset consists of 11,760 CEL files that survived the quality control procedures described in Section 2.2 and Section 2.3. We call this dataset of 11,760 CEL files as the “complete dataset”. To the best of our knowledge, this is the largest collection of microarray datasets for *Arabidopsis thaliana*. In this complete dataset, if we remove all the genes having IQR expression value less than 0.65 (as suggested by Aluru et al. (2012) [7]), only 13,384 genes remain for network construction. This is much less than the 15,495 genes that survived data filtering in the previous analysis of 3546 Affymetrix ATH CEL files reported in Aluru et al. (2012) [7].

As our dataset collection is inclusive of and more than thrice the size of the dataset used in Aluru et al. (2012) [7], it is reasonable to expect that the enlarged dataset should result in inclusion of more genes and discovery of more interactions. This conundrum of generating smaller networks with more data is due to the fact that gene interactions are tissue or process specific. The inclusion of a large number of experiments unrelated to the scope of a gene interaction can negatively affect its discovery. In addition, this may also lead to filtering out of a relevant gene in the network due to lack of sufficient dynamic range of expression over the entire dataset. As a consequence, simply enlarging the dataset and processing it as a whole does not lead to larger networks with more comprehensive discovery of novel gene interactions. We postulate that proper categorization of experiments is crucial to quality of gene network constructions, and demonstrate this as follows.

We classified the collection of 11,760 CEL files into tissue-specific and process-specific classes. We developed a two-step classification procedure. In the first step, we search for keywords related to different plant tissues and the physiological processes within the description and metadata available for each submission in the database. Based on the keywords identified, we made a preliminary partition of the 11,760 CEL files. In this preliminary assignment, one submission can be assigned to multiple categories. In the second step, we manually scanned the metadata available for each experiment and refine the preliminary classification further to resolve any error in the assigned categories. Table 2 shows the list of tissue and process categories we use for classification and the corresponding number of experiments and CEL files under each category.

### 2.7. Network Construction Methods

Though we focus primarily on pre-processing techniques in this paper, we briefly discuss network construction methods here because the effectiveness of the proposed pre-processing techniques is evaluated in the context of network construction. Reverse-engineering networks are a well-studied problem and many different methods with varying capabilities have been developed for network construction (for example, [15,16,17,18]).

For the purpose of evaluating our pre-processing methods, we use two different network-reverse engineering methods. In both of these methods, a correlation measure is evaluated for every pair of genes in the dataset, and correlations that are deemed significant are reported as edges in the output network. One uses the Pearson correlation coefficient as the correlation measure, while the other uses Mutual Information.

Pearson correlation coefficient (PCC) is a measure of linear relationship between any two variables of interest and it ranges from +1 to −1, where +1 indicates the strongest positive correlation, while −1 a strong negative correlation. We use the pcor function in R to compute PCC. After measuring PCC for every pair of genes, we use the method used by Mao et al. (2009) [8] to estimate the threshold above which a correlation can be considered significant.

Mutual Information (MI) between two random variables *x* and *y* is a measure of dependence between *x* and *y*. We use the TINGe software [19] to construct MI network. TINGe is a parallel gene network construction method that uses a B-Spline based method to estimate MI values [20] between every pair of genes and, then evaluates statistical significance using permutation testing. It also uses data processing inequality (DPI) to eliminate indirect relations between two genes. We refer the reader to [19] for further details on TINGe.

## 3. Results

We discuss data filtering and classification strategies under the context of gene network construction. First, in Section 3.1, we analyze the results of applying the data filtering procedure on the complete dataset. Then, in Section 3.2, we discuss the reasons for the rejection of genes in the complete dataset by the filtering procedure. We also describe our rationale for classification, and show how classification aids in our goal to include as many genes as possible without having to compromise on the stringency of data filtering procedures. Finally, in Section 3.3, we analyze the results of PCC and MI networks built using the tissue-specific and process-specific datasets.

### 3.1. Data Filtering Applied to a Complete Dataset

As noted earlier, if we eliminate in the complete dataset the genes with IQR values less than 0.65 (a threshold value suggested by Aluru et al. (2012) [7]), only 13,384 genes remain for network construction. Note that compared to Aluru et al. (2012) [7], which applied the same threshold to 3456 observations, we are 1027 genes short. When we select the IQR threshold using the histogram plot method described in Section 2.4, the number of genes that survive the IQR filtering increases to 18,806. Figure 3 shows the IQR histogram plot constructed for the complete dataset.

**Figure 4:**
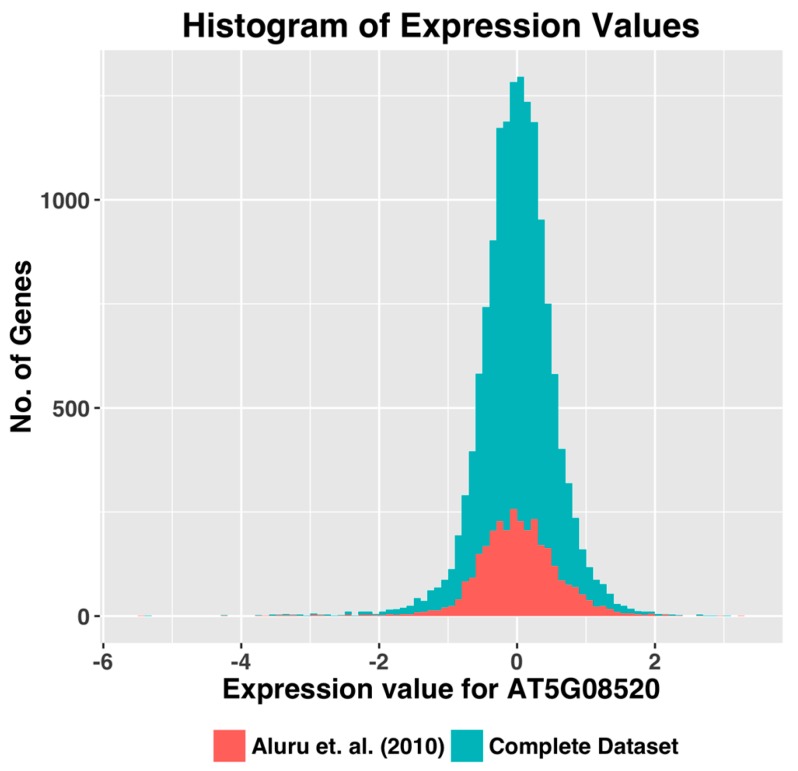
Expression value histogram compares the expression value distribution for the gene AT5G08520 between the dataset by [7] and our dataset.

Though the histogram method of selecting the IQR threshold is an improvement over using the threshold suggested by Aluru et al. (2012) [7], it still leaves out many of the ~22,000 genes that constitute the *Arabidopsis* *thaliana* genome. To determine why certain genes were excluded, we selected a few genes that were rejected by the data filtering procedure(s), and compared their expression level distribution. We observed that the relative expression levels of these genes remained low in a significant percentage of the experiments, although their expression levels were high in a few specific sets of experiments. As a result, these genes did not show the dynamic range of expression needed for co-expression analyses, even though the number of observations has increased. Figure 4 shows the histogram plot of expression values of one of the rejected genes.

### 3.2. Data Filtering Applied to Classfied Datasets

In eukaryotic organisms such as plants, gene expression levels vary according to tissue and/or the biological processes. Many genes are expressed only in those tissues and processes they actively participate in. Recent studies in maize have demonstrated this fact by constructing modules that show tissue-specific expression levels of groups of genes [21]. Therefore, in order to exploit our collection of datasets to the best possible extent, we categorized the experiments based on specific plant tissues or the biological process.

Table 3 shows the number of genes that survived the data filtering procedure for each of the tissue-specific and process-specific datasets.

Even though the number of genes covered by the individual classified datasets is in the range of 17,000–19,000, the total number of genes covered is 20,146, which exceeds the number of genes that pass the data filtering procedure for the complete dataset (Section 3.1). With the help of classification, we are able to increase the number of genes covered by the gene network construction from 15,498 in the previous work reported in Aluru et al. (2012) [7] to 20,146.

### 3.3. Networks Construction

We constructed both PCC and MI networks for each of the tissue-specific and process-specific datasets. We then constructed three union networks—the union of all tissue-specific networks (tissue union), the union of all process-specific networks (process union), and the union of all the networks (full union). We built these union networks from the constituent networks as a set union of their vertices and edges. Apart from the tissue-specific and process-specific networks, we also constructed networks for the complete dataset of 11,760 observations so as to compare the effect of the classification on reverse-engineering gene networks.

Table 4 shows the number of genes covered by both PCC and MI reverse-engineered networks using the complete dataset in two cases—(A) when the IQR threshold q is set to the value of 0.65 and (B) when the IQR threshold q is set using the histogram plot method described in Section 2.3. We use (A) to indicate the former case and (B) for the latter. Also shown in Table 4 are the results of large-scale genome networks constructed previously for *Arabidopsis thaliana* by [7,8,9].

For a given dataset, we observe that (i) all the genes included in the dataset may not be included in the network; and (ii) the number of genes included in the network depends upon the construction method. For example, in the case of dataset (B), the MI network fails to cover 200 genes present in the input dataset, whereas 14,866 genes are absent in the PCC network.

With respect to the second observation, we note that MI networks are able to include a larger number of genes compared to PCC networks. For example, in the case of dataset (B), the MI network has 14,866 genes more than the PCC network. The MI network also covers all the genes covered by the PCC network. The reason for this difference is that the PCC network measures only the linear correlations between the genes, whereas the MI network can measure both linear and non-linear relationships.

The results given in Table 4 show that in both the network construction methods, some genes are lost because of the nature of the correlation measures. Table 4 also demonstrates that the networks constructed with the 11,760 size complete dataset are, in fact, smaller than the corresponding MI and PCC networks constructed in earlier works by Aluru et al. (2012) [7] and Mao et al. (2009) [8], respectively, even though both [7,8] use significantly smaller datasets. These factors reinforce the need for pre-processing techniques that include as many genes as possible by carefully managing the diversity of large-scale datasets. We achieve this goal by means of classification, as the following results show.

Table 5 shows the network sizes of the PCC and MI networks of the classified datasets described in Table 3.

The tissue and process union of PCC networks has 7560 and 6858 genes, respectively. The full union PCC network has a total of 8429 genes and has 131,648 edges. In the case of the MI networks, the tissue and process union of the MI networks has 19,247 and 20,045 genes, respectively—significantly larger than the corresponding PCC union networks. The full union MI network has a total of 20,126 genes and 638,051 edges.

Since PCC networks can only identify linear relationships between the variables while MI networks can recover both linear and non-linear correlations, all the PCC networks include significantly fewer genes compared to the corresponding MI networks. Also, Table 3 and Table 5 show that the larger the number of genes in the input dataset, the larger the number of genes in the network. Most importantly, by making a union of PCC networks we are able to cover 4489 genes more than the network constructed for the complete dataset. We observe a similar behavior in the case of the full union MI network also, where the gain is 1320 genes.

## 4. Discussion

A stringent pre-processing pipeline precedes any analysis of microarray data, whether the goal is to identify differentially expressed genes or to learn gene networks. In Section 2.2 and Section 2.3 we discussed these standard pre-processing methods for quality control, normalization and data filtering used in network construction methods.

However, as we discussed in Section 2.4, Section 3.1 and Section 3.2, they prove to be insufficient for inferring genome-scale networks from a large collection of gene expression data. When the goal is to construct networks for eukaryotic organisms with tens of thousands of genes from a large dataset, the process of aggregation from multiple data sources introduces a limit in the utility of the downstream network construction methods. We observe this shortfall in Section 3.3 (Table 4) with the complete dataset. The data filtering procedure commonly used for the Affymetrix ATH Platform leaves us with only 18,806 genes (Section 3.1), which in turn leads to MI and PCC networks of sizes of 18,606 and 3940, respectively (Table 4).

On closer examination of genes rejected by data filtering (Figure 4), we found that they have a lower expression range in many experiments and are hence rejected. Contrary to expectations, we observed that the increase in the number of experiments does not automatically translate to an increase in the number of genes available for network construction, which in turn lead us to this fundamental insight: because of the spatial and temporal expression of genes in eukaryotic organisms, certain genes are actively expressed in only specific tissues and biological processes. Therefore, the activity of these genes becomes less recognizable when all the experiments are analyzed in aggregate.

Our solution to address this problem is to devise a classification procedure that assigns the datasets to the tissue-specific and process-specific categories described in Section 2.4. Each of these classified datasets is subjected to independent pre-processing runs. Though the number of genes covered by classified datasets is in the range of 17,000 to 19,000 (Table 3), 20,146 genes were covered in total (Section 2.6). By increasing the total coverage via classification, we are able to increase the number of genes covered by the largest union network to 20,045 (Section 3.3). To the best of our knowledge, this is the largest gene network constructed for *Arabidopsis thaliana* using only publicly available microarray data.

Though we describe our methods in the context of *Arabidopsis thaliana*, they are general in nature and can be applied to any organism. An interesting open question is whether, for a given dataset, the category names can be automatically derived from the data sizes and metadata available.

## 5. Conclusions

In this paper, we describe a method to pre-process large-scale microarray datasets, specifically in the context of reverse-engineer networks. Our novel pre-processing method led to the construction of the largest network from microarray datasets for *Arabidopsis thaliana*. We hope that the generality of our method enables the construction of genome-scale networks of other eukaryotic organisms of interest, and hence the discovery of novel interactions.

## Figures and Tables

**Table 1:** Microarray data collected from public databases. Columns show the list of databases, the number of experiments obtained from each database and the number of original CEL files that passed quality control.

Database	Experiments	CEL Files	QC Filtered
ArrayExpress	543	7923	848
GEO	83	1270	102
NASC	211	2859	387
AtGenExpress	44	1334	289
Total	881	13,386	1626

**Table 2:** Classification of microarray experiments. Columns show the classification basis, class, number of experiments assigned to the particular class, corresponding number of CEL Files.

Basis	Class	Experiments	CEL Files
Process	Chemical	75	808
	Development	190	2252
	Hormone	116	1806
	Light	64	1210
	Metabolism	214	1535
	Pathogen	69	1156
	Stress	153	2476
Tissue	Flower	69	764
	Leaf	279	4268
	Root	121	1939
	Seedling	379	5234
	Whole Plant	144	2359

**Table 3:** Data filtering results after classification, showing the number of genes that survive the filter.

Basis	Class	Genes
Process	Chemical	18,026
	Development	17,827
	Hormone	17,646
	Light	17,895
	Metabolism	17,989
	Pathogen	17,486
	Stress	19,041
Tissue	Flower	17,209
	Leaf	17,215
	Root	17,775
	Seedling	17,960
	Whole Plant	18,805

**Table 4:** Number of genes covered by reverse-engineered networks. Shows the sizes of networks constructed from complete dataset when (A) IQR threshold *q* = 0.65 and (B) *q* is selected from the histogram plot. Also shown in the table are the sizes of networks constructed by [7,8,9].

Network	Size of Input Datset	Genes in Input Dataset	Genes in Network
PCC with dataset (A)	11,760	13,384	2670
MI with dataset (A)	11,760	13,384	13,181
PCC with dataset (B)	11,760	18,806	3940
MI with dataset (B)	11,760	18,806	18,606
MI network by [7]	3137	15,578	15,495
PCC network by [8]	1094	16,293	6206
GGM network by [9]	2045	NA ^1^	6760

^1^ [9] constructs the GGM network by randomly sampling 2000 genes at a time. NA ^1^: Not Applicable; PCC: Pearson correlation coefficient; MI: Mutual Information; GGM: Gaussian Graphical Model.

**Table 5:** Network statistics for all the classified datasets.

Basis	Network	Pearson Correlation Coefficient Networks		Mutual Information Networks	
		Vertices	Edges	Vertices	Edges
Process	Chemical	2553	51,934	17,355	109,837
	Development	2284	53,890	17,813	90,238
	Hormone	1696	17714	17,598	98,954
	Light	3575	175,171	17,877	99,671
	Metabolism	4190	302,844	18,026	90,564
	Pathogen	2494	85,468	17,406	115,085
	Stress	4078	919,149	19,036	182,545
Tissue	Flower	3712	82,947	16,594	122,866
	Leaf	3073	119,432	17,210	73,168
	Root	2549	141,982	17,768	103,778
	Seedling	4156	314,204	17,947	82,494
	Whole Plant	5152	1,054,976	18,797	136,115

## Abbreviations

The following abbreviations are used in this manuscript:

AGI	Arabidopsis Gene Identifier
GEO	Gene Expression Omnibus
GGM	Gaussian Graphical Model
IQR	Inter-quartile range
MI	Mutual Information
NASC	Nottingham Arabidopsis Stock Centre
NCBI	National Center for Biotechnology Information
NUSE	Normalized Unscaled Standard Errors
PCC	Pearson Correlation Coefficient
RLE	Relative Log Expression
TAIR	The Arabidopsis Information Resource
